# Hyperspectral Imaging and Selected Biological Control Agents for the Management of Fusarium Head Blight in Spring Wheat

**DOI:** 10.3390/plants12203534

**Published:** 2023-10-11

**Authors:** Martin E. G. Rieker, Maximilian A. Lutz, Abbas El-Hasan, Stefan Thomas, Ralf T. Voegele

**Affiliations:** Department of Phytopathology, Institute of Phytomedicine, Faculty of Agricultural Sciences, University of Hohenheim, 70599 Stuttgart, Germany; maxi.lutz98@gmail.com (M.A.L.); aelhasan@uni-hohenheim.de (A.E.-H.); stefan.thomas@uni-hohenheim.de (S.T.); ralf.voegele@uni-hohenheim.de (R.T.V.)

**Keywords:** Fusarium head blight (FHB), wheat, hyperspectral imaging (HSI), biocontrol, biological control agents (BCAs)

## Abstract

*Fusarium* spp. are important pathogens on cereals, capable of causing considerable yield losses and significantly reducing the quality of harvested grains due to contamination with mycotoxins. The European Union intends to reduce the use of chemical-synthetic plant protection products (csPPP) by up to 50% by the year 2030. To realize this endeavor without significant economic losses for farmers, it is crucial to have both precise early detection of pathogens and effective alternatives for csPPP. To investigate both the early detection of Fusarium head blight (FHB) and the efficacy of selected biological control agents (BCAs), a pot experiment with spring wheat (cv. ‘Servus’) was conducted under semi-field conditions. Spikes were sprayed with different BCAs prior to inoculation with a mixture of *F. graminearum* and *F. culmorum* conidia. While early detection of FHB was investigated by hyperspectral imaging (HSI), the efficiency of the fungal (*Trichoderma* sp. T10, *T. harzianum* T16, *T. asperellum* T23 and *Clonostachys rosea* CRP1104) and bacterial (*Bacillus subtilis* HG77 and *Pseudomonas fluorescens* G308) BCAs was assessed by visual monitoring. Evaluation of the hyperspectral images using linear discriminant analysis (LDA) resulted in a pathogen detection nine days post inoculation (dpi) with the pathogen, and thus four days before the first symptoms could be visually detected. Furthermore, support vector machines (SVM) and a combination of LDA and distance classifier (DC) were also able to detect FHB symptoms earlier than manual rating. Scoring the spikes at 13 and 17 dpi with the pathogen showed no significant differences in the FHB incidence among the treatments. Nevertheless, there is a trend suggesting that all BCAs exhibit a diminishing effect against FHB, with fungal isolates demonstrating greater efficacy compared to bacterial ones.

## 1. Introduction

**Figure 1:**
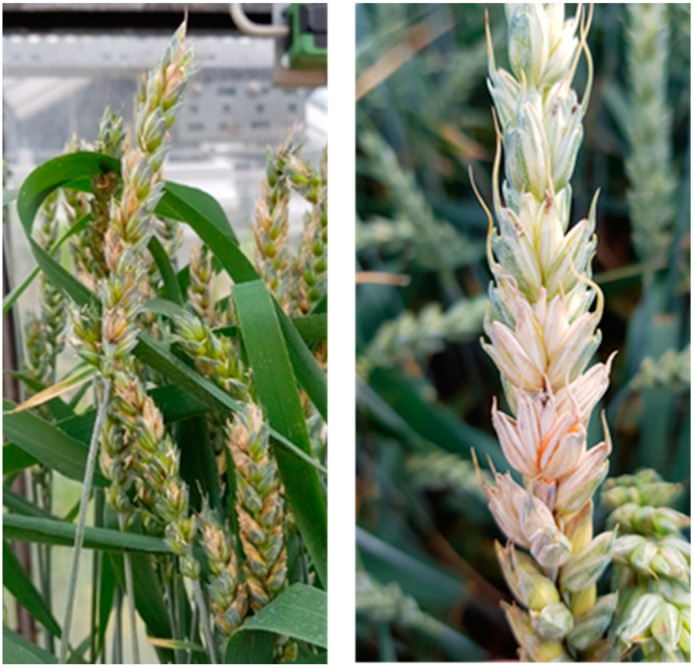
Symptoms of Fusarium head blight (FHB) on infected spikes (**left**) and a highly infected spike with pink/orange-colored spore-producing mycelium (**right**).

Wheat is one of the most important crops with the largest cultivated area of all crops worldwide [1]. In 2020, this was 220 Mio. hectares [2]. It has a good baking quality and is ideally suited for human nutrition and as livestock fodder because in addition to carbohydrates, grains contain high amounts of proteins, fats, minerals, vitamins and fiber [1,3,4]. Numerous pathogens threaten wheat production. Among the most devasting fungal pathogens are members of the genus *Fusarium*. Especially, *F. graminearum*, *F. culmorum* and *F. avenaceum* cause foot rot and spike (head) blight on small-grain cereals [5,6]. Fusarium head blight (FHB), for instance, can occur regularly on all important small-grain cereals, including wheat, rye, barley, oats and triticale [7]. Ascospores or conidia of *Fusarium* spp. are transmitted by wind and water and infect the host through the flowers [8]. Thus, cereals are particularly susceptible in the phase between spike emergence and the end of anthesis [9]. Infected spikelets turn pale brown to white from the base and become dark brown as the infection progresses [6]. As the fungus develops into the rachis, the water and nutrient supply is interrupted, resulting in partial or total depletion of spikes (Figure 1) [5,6,9]. High economical losses are due to reduced yield and a remarkable decline in quality. This includes a diminished baking quality, decreased germination rate and elevated mycotoxin levels (e.g., trichothecenes, zearalenone) in the harvested kernels [9,10].

Fungal plant pathogens can affect the quantity and quality of a wide variety of crops. As the European Union intends to reduce pesticide use by 50% by 2030, more sustainable crop protection alternatives have to be established in order to maintain the yield and quality of the crops [11]. This includes the use of optical methods to detect pathogen infection at an early stage, along with implementing biological plant protection products as substitutes for chemical-synthetic fungicides [12,13]. Precise and reliable pathogen detection and quantification provide a fundamental tool for decision-making on the application of plant protection measures [14,15]. Traditional visual monitoring is the most commonly used method for evaluating plant diseases [16]. In addition, molecular detection methods, such as PCR, are also among the standard methods, as they have a high sensitivity and precision [17]. However, both methods come with several disadvantages. Laboratory tests are destructive, invasive and require transport of samples. Meanwhile, visual scoring is not only time-intensive but also susceptible to errors [15]. In addition, visual monitoring can only detect infections once symptoms and damage have become visible [18]. In contrast, an alternative option is hyperspectral imaging (HSI), which uses spectral information of the visible (VIS) and near-infrared (NIR) range [12,18]. HSI systems measure the reflectance of several hundred very narrow spectral bands in a broad spectrum, allowing an accurate spectral resolution [18]. Compared to optical monitoring, pathogen detection can be performed earlier with HSI, as even small changes in the metabolism or structure of the plant resulting from an infection can be detected before symptoms become visible [15,19]. Numerous studies already showed that HSI successfully enables detection of various pathogens on living plants and harvested crops [20,21,22]. Bauriegel et al. [23] succeeded in detecting FHB in different developmental stages of wheat plants under laboratory conditions using HSI. Barbedo et al. [24] were also able to determine what wheat grains are affected by FHB with 91% accuracy using HSI. In addition, early detection and quantification of infection in other pathosystems such as barley—brown rust and grapefruit—citrus canker was possible by using HSI [20,22].

Early detection of FHB infection must be followed by appropriate plant protection measures. In conventional farming, chemical-synthetic fungicides with active ingredients such as azoles are used in a large scale to control FHB, with a relatively high efficiency. One more sustainable control measure is the use of biological control agents (BCAs), which are coming into focus as an eco-friendly alternative for chemical-synthetic plant protection products [13,25,26]. BCAs, including bacteria, fungi, yeasts or viruses, achieve their suppressive effects through one or a combination of several modes of action, e.g., competition for nutrients or space, antibiosis, induction of plant resistance and hyper-parasitism [27,28]. BCAs should effectively establish themselves on the plant, thereby diminishing pathogen infection, inhibiting their growth, propagation and toxin accumulation [25].

The objective of this study was to evaluate how early and reliably FHB can be detected in wheat under laboratory conditions using HSI. HSI results were compared with the results of conventional visual monitoring. Furthermore, selected microorganisms were tested for their efficiency as BCAs applied against FHB in wheat within semi-field conditions.

## 2. Results

### 2.1. Spectral Signatures of Different Areas on the Spikes

For a successful early detection of FHB in wheat by using HSI, it was first necessary to identify wavelength windows with clear differences in reflectance between non-infected, healthy and infected symptomatic spike areas. Additionally, differences from symptomatic areas to maturity as well as to flowers should be identified.

**Figure 2:**
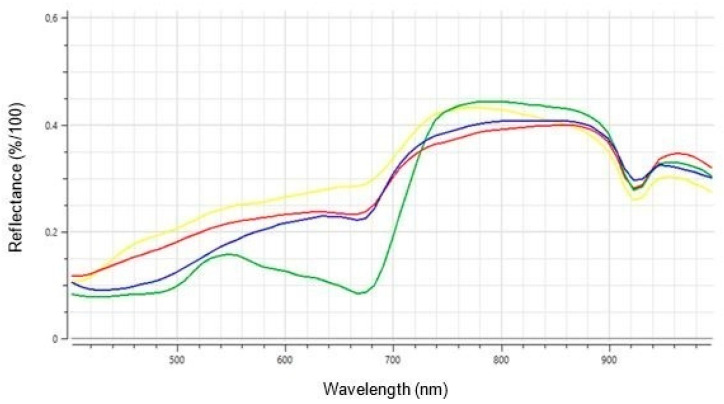
Spectral signatures of the classes “Flower” (yellow), “Symptom” (red), “Healthy” (green) and “Maturity” (blue) in the spectrum from 400 to 1000 nm of spikelet tissue.

Figure 2 shows the reflectance of the mean values of the manually generated classes on a scale from 0 to 1 as a function of wavelength, which means 0% to 100% of the reflected light. Reflectance values were normalized to a dark reference as value 0 and a white reference as value 1. The wavelength range covered the VIS spectrum from 400 to 1000 nm and part of the NIR range. The average reflectance values of the classes ranged from 8% to 45%. There were clear differences between the four classes, especially for the wavelength range of 400–800 nm. Healthy, symptomless spikelet tissue had the lowest reflectance in the lower wavelength range, up to about 720 nm. It showed a higher reflectance than the other classes for the interval of 740–900 nm. In the range of 550–700 nm, the difference was particularly pronounced. The curves of “Flower”, “Symptom” and “Maturity” did not differ much in comparison. However, there were differences as well. The class “Flower” showed the highest reflectance values for the wavelength range of 450–740 nm. “Symptom” and “Maturity” primarily differed in the range of 420–600 nm, where infected, symptomatic tissue had higher reflectance values than senescent spikelet tissue. The reflectance values of classes “Maturity” and “Healthy” significantly differed from each other in the wavelength range up to 900 nm.

### 2.2. Early Detection of FHB by HSI

Based on the 15-day measurement series from 6 to 20 days post inoculation (dpi) with FHB, early detection of the infection was tested using these hyperspectral images. Different supervised and unsupervised classification methods were used, so that the day of their first detection was compared with the day when FHB symptoms could be detected by visual assessment for the first time. This was performed for the treatments “Control + *Fusarium*”, “T10 + *Fusarium*”, T16 + *Fusarium*” and “T23 + *Fusarium*”.

**Figure 3:**
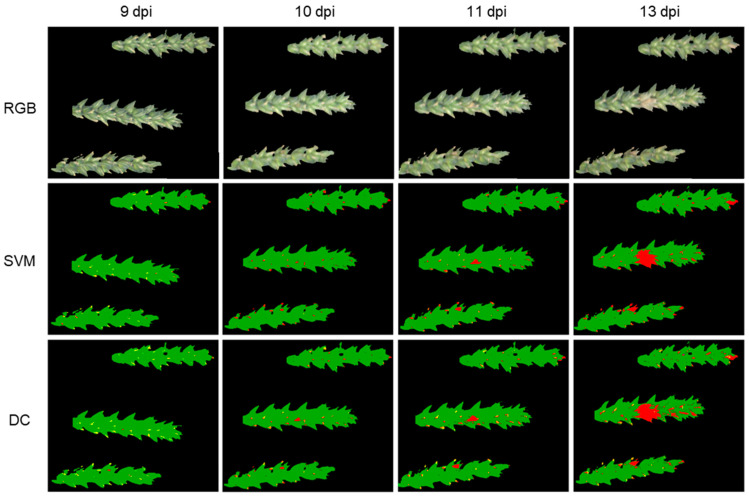
Test of early detection of FHB infection with the algorithms support vector machines (SVM) and distance classifier (DC), performed with spikes 8–10 of the treatment “Control + *Fusarium*” as an example at 9, 10, 11 and 13 days post inoculation (dpi). Within the columns, the day of recording is the same. Pixels were classified into different classes by machine learning methods and color-coded: Healthy (green), Flowers (yellow) and Symptomatic (red). In comparison, the pseudo-RGB image shows the extent to which FHB symptoms could be detected by visual assessment.

In contrast to support vector machines (SVM), distance classifier (DC) was preceded by a linear discriminant analysis (LDA). In the following text, when the term DC is used, it refers to this combination. The classifications of SVM and DC of “Control + *Fusarium*” were contrasted with the RGB images for the days 9, 10, 11 and 13 dpi (Figure 3). A visual identification of FHB symptoms was first possible with RGB images 13 dpi. At 11 dpi, the first color changes were already visible; however, they were not sufficient for a clear detection. Both SVM and DC often made misclassifications for flowers. While at 9 dpi these areas were mostly correctly color-coded by both methods and thus assigned to the class “Flower”, they were often classified as symptomatic for the following images at 10, 11 and 13 dpi. With SVM, infection could already be detected at 11 dpi. Infection sites could be identified on the middle and lower spikes in Figure 3. These can be distinguished from the incorrectly assigned red-colored flower regions by their size. As SVM at 13 dpi shows, the infected area at the tip of the upper spike was also already correctly identified at 11 dpi. However, due to its still small size, this area could not yet be distinguished from the mislabeled flowers at this time. The first symptoms were detected using DC starting at 10 dpi. Thus, DC could detect infected areas one day before SVM and three days before a visual identification.

**Figure 4:**
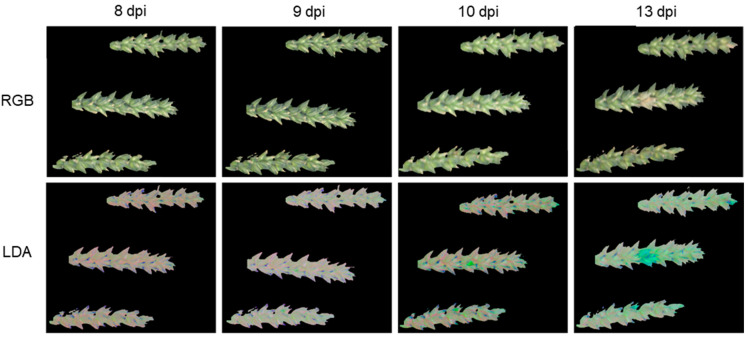
Comparison of spikes 8–10 of the treatment “Control + *Fusarium*” at 8, 9, 10 and 13 dpi as pseudo-RGB images and as false color images, representing the linear discriminant analysis (LDA) classification. Within the columns, the day of recording is the same. In the LDA classifications, healthy tissue is colored in pink and light green depending on the shadow cast, flowers are colored in dark blue and symptomatic areas with FHB are first colored in light green and later in time in turquoise to blue.

In addition to the combination of LDA and subsequent DC, pathogen detection was also tested for the LDA without subsequent DC. A comparison of the pseudo-RGB images and LDA for the variant “Control + *Fusarium*” is shown in Figure 4. The “Three-Column Mode” that was used as image output for the LDA uses information from all three dimensions simultaneously for the false color representation. Healthy spikes are colored pink or dull light green, flowers are shown in purple and *Fusarium*-infected areas are colored light green and became recognizable at 9 dpi. From 10 dpi on, these areas were clearly distinguishable from healthy tissue. As infection progressed, the color of the infected areas changed to turquoise and blue. In contrast to SVM and DC, flowers were not classified as symptoms by LDA.

**Figure 5:**
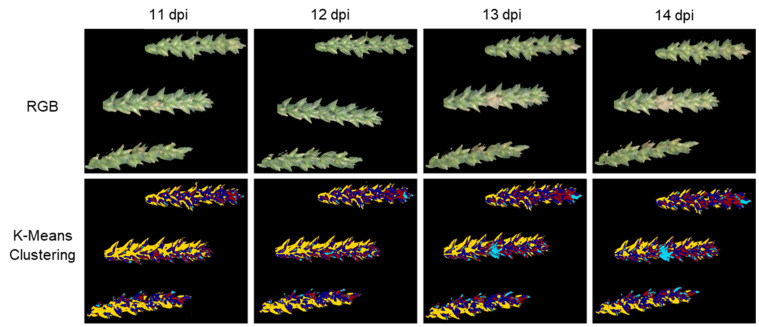
Comparison of spikes 8–10 of the treatment “Control + *Fusarium*” at 11, 12, 13 and 14 dpi as pseudo-RGB images with the classification of K-Means clustering with ClusterSize 4. The unsupervised algorithm color-coded healthy tissue in yellow, red or blue depending on the light conditions. Both the infected areas and a large part of the flowers are color-coded in light blue.

K-Means clustering, an unsupervised method, was also tested (Figure 5). With K-Means clustering, pathogen detection was possible in the first 13 dpi. In addition to the correctly identified infected areas, other areas were also colored light blue, which were not classified as such by the other classification methods as well as by the visual assessment of images taken later in the measurement series.

Early pathogen detection of the different classification methods was not only tested for the treatment “Control + *Fusarium*”. However, only the results of this variant are shown because pathogen identification occurred earliest here for all classification methods.

### 2.3. Validation of Hyperspectral Imaging by Molecular Analysis

Using PCR as a molecular detection method, sampled spikelets were tested for the presence of FHB. This was necessary to check whether areas detected as symptomatic/infected by the classification methods were actually infected by FHB.

**Figure A1:**
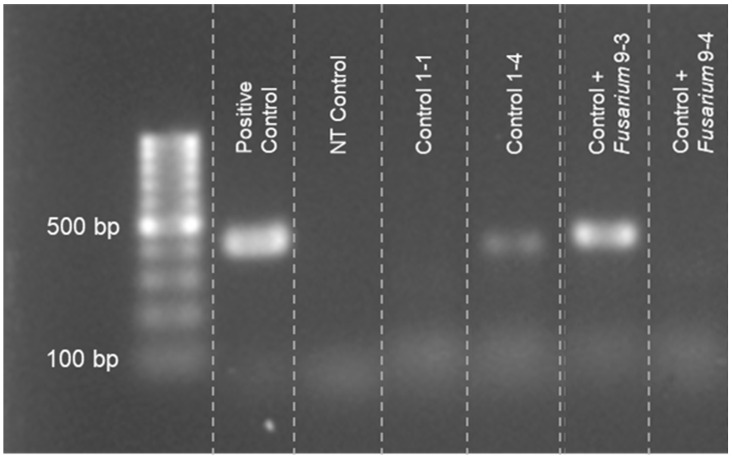
Gel electrophoresis image with spikelet samples of the treatments “Control” and “Control + *Fusarium*”. The PCR was carried out with the primer pair TEF-Fu3 f and TEF-Fu3 r.

The result of the gel electrophoresis, which was carried out with the PCR products, is shown for some samples in Figure A1. As can be seen in the positive control, DNA of *Fusarium* spp. in the sample was characterized by a band at approximately 400–500 base pairs (bp). The sample “Control 1-4” had a weak but clearly visible band at this level. This could either have been caused by contamination, or the corresponding spikelet was actually infected with FHB by natural infection. Except for “Control + *Fusarium* 9-4”, all samples from the variants inoculated with a *Fusarium* spore suspension showed a band in the range of 400–500 bp.

### 2.4. Quantitative Evaluation of the Classification Methods

The supervised machine learning methods, SVM and DC, were tested for their reliability and precision. For this purpose, their classification of the pixels into the four classes was compared with a reference. For the reference, the pixel classification of the pseudo-RGB into the classes was performed manually by the experts M.L. and M.R. The comparisons of the classifications are listed as the number of pixels per class and the proportion of total pixels. K-Means clustering could not be evaluated this way because as an unsupervised classification method, it does not classify the pixels into the specified classes. Thus, a quantitative comparison with the reference is not possible.

As example, the comparison for the variant “Control + *Fusarium*” is shown in Table 1. The treatment “Control + *Fusarium”* had a total of 55,110 pixels. With 50,438 pixels, the reference allocated 91.52% to the “Healthy” class. With 49,584 pixels (89.97%) for SVM and 47,921 pixels (86.96%) for DC, the proportion was lower for the supervised methods. In the manual classification, 1007 pixels (1.86%) were declared as flowers. Again, the values of SVM (196 pixels, 0.36%) and DC (747 pixels, 1.36%) were lower. With regards to maturity, this tendency was again evident. With 181 pixels (0.33%), the reference had a larger number of pixels assigned than SVM (3 pixels, 0.01%) or DC (12 pixels, 0.02%). The classification of the areas infected with FHB did not show this trend. There, with 3484 allocated pixels (6.32%), the reference had a smaller share than SVM (5327 pixels, 9.67%) or DC (6430 pixels, 11.67%).

**Figure 6:**
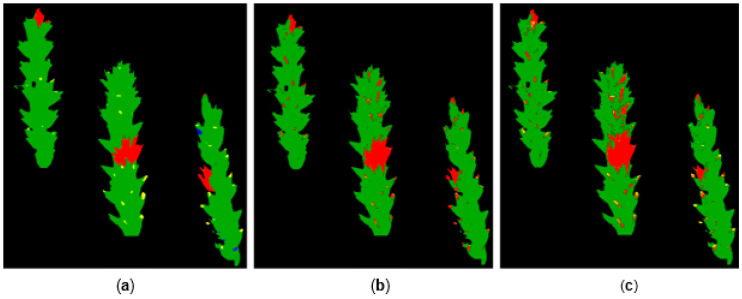
Comparison of classification methods for spikes 8–10 of the treatment “Control + *Fusarium*” at 13 dpi: (**a**) reference, manually classified pseudo-RGB by an expert, (**b**) SVM and (**c**) DC. Pixels with healthy tissue are color-coded green, flowers yellow, maturity blue and FHB symptoms red. Symptomatic areas were scored similarly in all three sub-images. There were differences in some areas, which were marked as flowers (yellow) in (**a**). These were partially classified as symptoms by SVM and DC.

In addition, the manual classification of spike areas and classifications by SVM and DC were also compared with each other graphically. Figure 6 shows the classification of spikes 8–10 of the treatment “Control + *Fusarium*”. The three symptomatic areas visible in sub-image (a) were also identified by SVM and DC, where similarly sized areas were also classified as infected with FHB. However, both algorithms also classified pixels as symptoms, which were identified as flowers in the reference.

### 2.5. Visual Monitoring of FHB Symptoms

**Figure 7:**
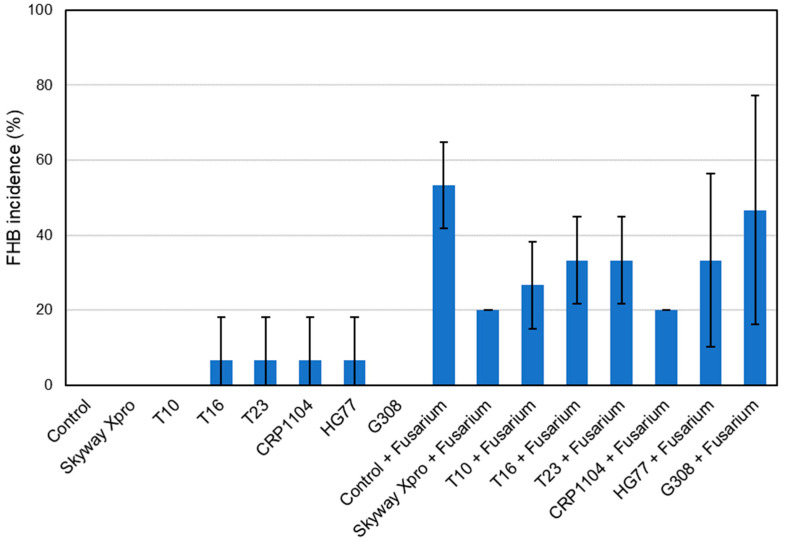
FHB incidence (%) of spring wheat spikes (cv. ‘Servus’) after different treatments: Fungicide Skyway Xpro, *Trichoderma* sp. T10, *T. harzianum* T16, *T. asperellum* T23, *C. rosea* CRP1104, *B. subtilis* HG77 and *P. fluorescens* G308. Monitoring was performed at 13 dpi with a mixture of *F. graminearum* and *F. culmorum*. Values represent the mean ± standard deviation (*n* = 3).

**Figure 8:**
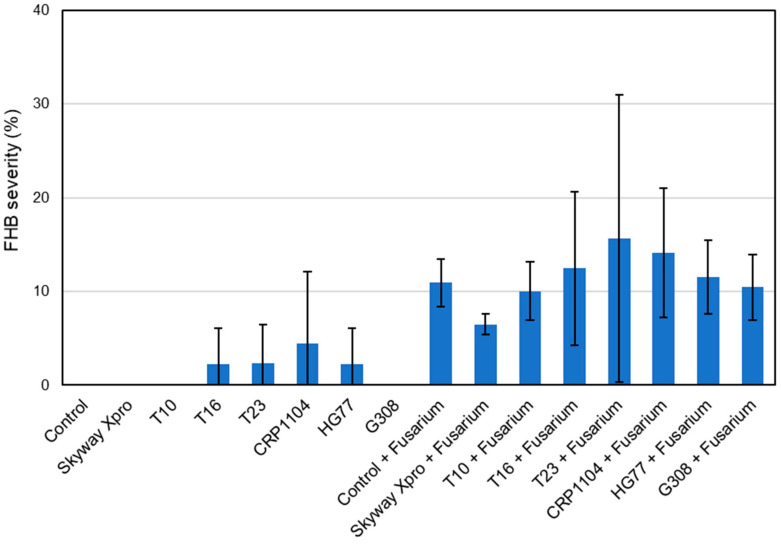
FHB severity (%) of spring wheat spikes (cv. ‘Servus’) after different treatments: Fungicide Skyway Xpro, *Trichoderma* sp. T10, *T. harzianum* T16, *T. asperellum* T23, *C. rosea* CRP1104, *B. subtilis* HG77 and *P. fluorescens* G308. Monitoring was performed at 13 dpi with a mixture of *F. graminearum* and *F. culmorum*. Values represent the mean ± standard deviation (*n* = 3).

**Figure 9:**
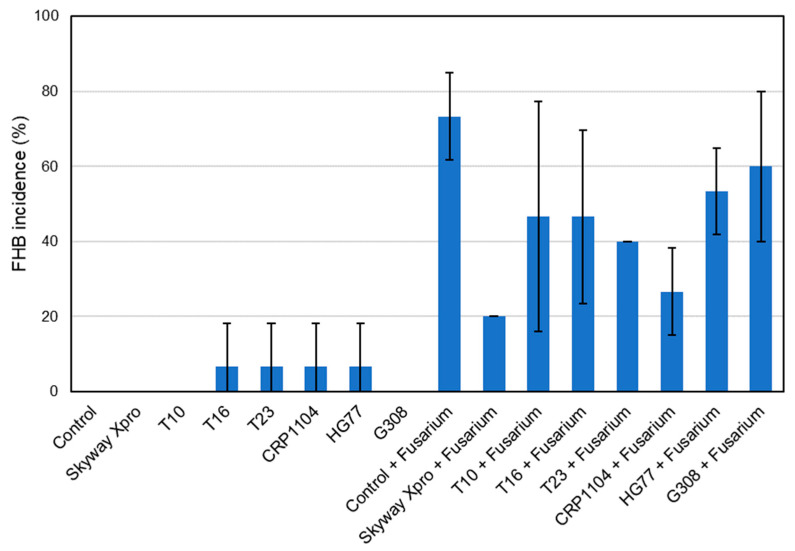
FHB incidence (%) of spring wheat spikes (cv. ‘Servus’) after different treatments: Fungicide Skyway Xpro, *Trichoderma* sp. T10, *T. harzianum* T16, *T. asperellum* T23, *C. rosea* CRP1104, *B. subtilis* HG77 and *P. fluorescens* G308. Monitoring was performed at 17 dpi with a mixture of *F. graminearum* and *F. culmorum*. Values represent the mean ± standard deviation (*n* = 3).

**Figure 10:**
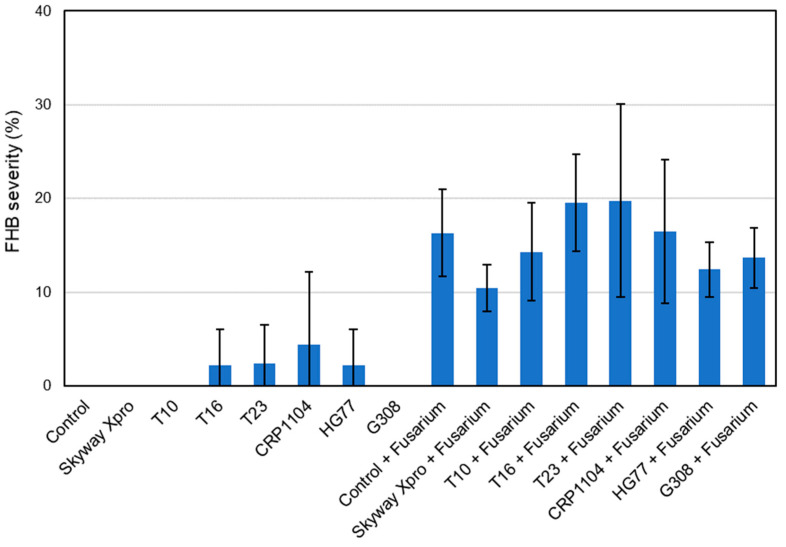
FHB severity (%) of spring wheat spikes (cv. ‘Servus’) after different treatments: Fungicide Skyway Xpro, *Trichoderma* sp. T10, *T. harzianum* T16, *T. asperellum* T23, *C. rosea* CRP1104, *B. subtilis* HG77 and *P. fluorescens* G308. Monitoring was performed at 17 dpi with a mixture of *F. graminearum* and *F. culmorum*. Values represent the mean ± standard deviation (*n* = 3).

Statistical analysis of the monitoring data based on the evaluation of pots 1–3 showed no significant differences between the treatments at both 13 and 17 dpi. Therefore, the results are only described, and tendencies are provided. For FHB incidence as well as FHB severity, the mean values of the treatments are shown in Figure 7, Figure 8, Figure 9 and Figure 10. These means were calculated from the three values per treatment evaluated by visual monitoring.

#### 2.5.1. FHB Incidence and FHB Severity 13 dpi

For the treatments “Control”, “Skyway Xpro”, “T10” and “G308”, no infection with FHB could be visually observed, as in these treatments no artificial inoculation with the pathogen was performed and obviously, there was no natural infection of the evaluated spikes by air-transmitted spores of *Fusarium* spp. (Figure 7). The treatment “Control + *Fusarium*”, which was only inoculated with the mixture of *Fusarium* spores, showed the most FHB symptoms, resulting in the highest FHB incidence (53.33%). “T16”, “T23”, “CRP1104” and “HG77” all had the same FHB incidence of 6.67%, which might be due to a naturally occurring FHB infection of some spikes of these treatments. Among the treatments inoculated with *Fusarium,* “Skyway Xpro + *Fusarium*” (20.00%) and “CRP1104 + *Fusarium*” (20.00%) showed the lowest FHB incidence. Application of the bacterial antagonist G308 led to an FHB incidence of 46.67%, which was the least effective of all BCA treatments.

FHB severity for “Control”, “Skyway Xpro”, “T10” and “G308” was 0% since no symptoms were detected on the spikes of these treatments at 13 dpi (Figure 8). “T16”, “T23” and “HG77” had low values for FHB severity (T16, HG77: 2.22%; T23: 2.38%), which were almost the same and presumably resulted from a natural infection with FHB. Among the FHB-inoculated variants, “T23 + *Fusarium*” had the highest FHB severity of 15.64% and a very high standard deviation. “Control + *Fusarium*” had 10.92% FHB severity. The lowest FHB severity among the *Fusarium*-inoculated treatments could be observed for “Skyway Xpro + *Fusarium*” (6.50%).

**Figure A2:**
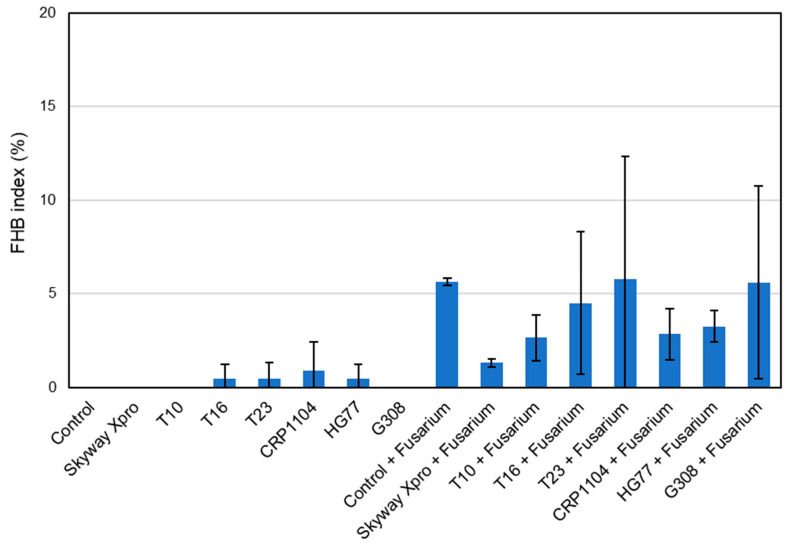
FHB index (%) of spring wheat spikes (cv. ‘Servus’) after different treatments: Fungicide Skyway Xpro, *Trichoderma* sp. T10, *T. harzianum* T16, *T. asperellum* T23, *C. rosea* CRP1104, *B. subtilis* HG77 and *P. fluorescens* G308. Monitoring was performed at 13 dpi with a mixture of *F. graminearum* and *F. culmorum*. The FHB index was calculated with the FHB incidence and FHB severity. Values represent the mean ± standard deviation (*n* = 3).

Figure A2 shows the FHB index for the evaluation at 13 dpi. Since this value is composed of FHB incidence and FHB severity, the treatments “Control”, “Skyway Xpro”, “T10” and “G308” had an FHB index of 0%. “T16”, “T23” and “HG77” had similar values, which were slightly lower than that of “CRP1104” (0.89%). The treatment “T23 + *Fusarium*” had the most symptoms and, therefore, with 5.78%, the highest FHB index of all treatments. In comparison, “Control + *Fusarium*” (5.63%) and “G308 + *Fusarium*” (5.59%) showed only slightly lower values. For “Skyway Xpro + *Fusarium*” (1.30%), the lowest FHB index among the treatments inoculated with FHB could be observed.

#### 2.5.2. FHB Incidence and FHB Severity 17 dpi

At 17 dpi, FHB incidence for the four treatments, “Control”, “Skyway Xpro”, “T10” and “G308”, was still the same (0%) (Figure 9). “T16”, “T23”, “CRP1104” and “HG77” also remained at 6.67%. For the treatment “Skyway Xpro + *Fusarium*” (20.00%), there was no increase of symptomatic spikes. For all the other variants that were inoculated with the pathogen, FHB incidence increased compared to that at 13 dpi. Among the fungal BCAs, the spikes treated with CRP1104 before inoculation with FHB had the lowest FHB incidence of 26.67%. The highest FHB incidences were observed in the two treatments testing the bacterial BCAs, “HG77 + *Fusarium*” (53.33%) and “G308 + *Fusarium*” (60.00%). Only “Control + *Fusarium*” (73.33%) still showed more FHB infections.

The FHB severity of all treatments not inoculated with FHB did not change from the first to the second monitoring date (Figure 10). The values for the treatments inoculated with FHB increased, as there was a spread of the infection within the spikes. The highest FHB severity was found for “T23 + *Fusarium*” with 19.77%, while “T16 + *Fusarium*” (19.51%) was slightly lower. Thus, the FHB severities of both treatments were higher than that of “Control + *Fusarium*”. There, an average of 16.32% of the spikelets were infected with FHB. The value for “Skyway Xpro + *Fusarium*” (10.43%) was the lowest of all *Fusarium*-inoculated treatments. The two bacterial variants, “HG77 + *Fusarium*” (12.41%) and “G308 + *Fusarium*” (13.67%), could reduce the FHB severity to a greater extent than the fungal ones.

**Figure A3:**
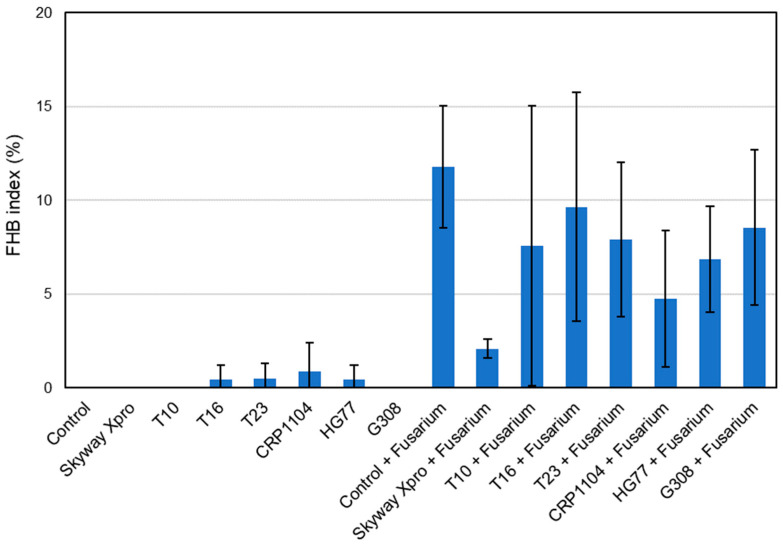
FHB index (%) of spring wheat spikes (cv. ‘Servus’) after different treatments: Fungicide Skyway Xpro, *Trichoderma* sp. T10, *T. harzianum* T16, *T. asperellum* T23, *C. rosea* CRP1104, *B. subtilis* HG77 and *P. fluorescens* G308. Monitoring was performed at 17 dpi with a mixture of *F. graminearum* and *F. culmorum*. The FHB index was calculated with the FHB incidence and FHB severity. Values represent the mean ± standard deviation (*n* = 3).

The FHB index for 17 dpi monitoring is shown in Figure A3. “Control”, “Skyway Xpro”, “T10” and “G308” had an FHB index of 0%. Treatments “T16” (0.44%), “T23” (0.48%) and “HG77” (0.44%) had similar values for the FHB index and were lower than “CRP1104” (0.89%). The highest FHB index was calculated for “Control + *Fusarium*” (11.79%). Among the treatments containing an inoculation with *Fusarium* spp., for “Skyway Xpro + *Fusarium*” (2.09%), the lowest FHB index was found. Comparing the treatments with BCAs and *Fusarium*, “T16 + *Fusarium*” (9.66%) had the highest value, while “CRP1104 + *Fusarium*” was the lowest, with 4.74%. Thus, the FHB index remained the same for the treatments not inoculated with *Fusarium* spp., while it increased for the other treatments with *Fusarium* inoculation.

## 3. Discussion

### 3.1. Spectral Signatures for FHB Identification

For identification of the spectral signatures by the fluxTrainer, the mean value of the pixels assigned to the classes as training data was used. Therefore, it was necessary to allocate a sufficient spike area to the classes to obtain representative values. This was a challenge for the class “Maturity”. Although spike areas from the last day of the measurement series (20 dpi) were used, the transition from healthy to senescent tissue was smooth. Thus, only clearly matured areas were selected.

The reflectance values of the classes “Healthy”, “Flower”, “Maturity” and “Symptom” were located between 8% and 45% (Figure 2), and thus in the same range as those published by Zhang et al. [29] for healthy wheat spikes and for different stages of FHB infection. The shapes of the curves in Figure 2 are also very similar to the spectral signatures of Zhang et al. [29]. Both showed clear differences between healthy spikes and areas with FHB symptoms. In the VIS range up to 720 nm, the non-FHB-infected tissue had a lower reflectance than areas infected with FHB. This relation was reversed for the neighboring NIR spectrum of 720–900 nm. Particularly clear differences were found in the wavelength ranges of 450–500 nm and 600–700 nm. In the spectrum of 600–700 nm, a distinct effect of the pathogen on the photosynthetic apparatus was seen. In case of infection, only a smaller proportion of irradiated light can be absorbed by the plant, which leads to a higher reflection compared to healthy plant tissue. Zhang et al. [29] reported a destruction of chloroplasts in the course of infection and thus a reduced amount of chlorophyll. The spectral signatures of the “Healthy” class show low reflectance values for blue (450–500 nm) and red (600–720 nm) light, the wavelengths used for photosynthesis (summarized by Kior et al. [30]). In between is the “green peak” [31]. Matured spikes as well as flowers and areas infected with FHB did not show this characteristic curve shape. Later, the tissue died off in all three cases and, therefore, had lower amounts of chlorophyll and carotenoids compared to healthy, not yet matured plant tissue [32].

### 3.2. Early Detection of FHB by HSI

Numerous pathogens can already be detected and identified by HSI [15]. Of particular interest for agricultural practice is an early detection. How much yield and quality losses occur is significantly determined by the time an infection is detected and suitable countermeasures are initiated. Pirgozliev et al. [33] showed that a fungicide must be applied close to the time of infection to reduce the amount of FHB infection, as well as deoxynivalenol (DON) concentration, in the harvested kernels.

Early detection of FHB infection was tested for all treatments inoculated with FHB. The first visible FHB symptoms appeared during the hyperspectral measurement series. On the day a pathogen infection is initially detected by a classification algorithm, usually only a few pixels of the infected area are assigned to the class “Symptom”. Thus, SVM and DC wrongly assigned a part of the flowers to the class “Symptom” due to misclassification (Figure 3). While at 9 dpi flowers were mostly correctly assigned to the class “Flower” by both methods, they were often classified as symptomatic for the following images at 10, 11 and 13 dpi. One explanation could be that training data for the class “Flower” were generated using images recorded at 9 dpi but the spectral signature of flowers changed within the measurement series over time, leading to misclassifications by SVM and DC due to the disparity with the selected training data. While possible to distinguish via comparison with later measurement points in the laboratory setup, in practical applications, this comparison is not possible or helpful. While the FHB-infected spike areas, which could later also be recognized visually, were already correctly detected at 10 dpi by SVM, they could first be clearly differentiated from misclassifications at 11 dpi.

K-Means clustering, as an unsupervised method, showed the latest initial FHB detection of all tested classification methods (Figure 5). The infection was noticed earliest on the same day, as with visual observation. As a reason for the comparatively late pathogen detection, K-Means clustering, in contrast to SVM and DC, does not access the training data of the previously created classes but subdivides the pixels into clusters exclusively based on structures in the raw data [34]. The supervised methods SVM and DC divided the pixels into the classes “Healthy”, “Symptom”, “Flower” and “Maturity” based on the training data (Figure 3).

Compared to the visual evaluation, the LDA without subsequent DC enabled the earliest detection of the FHB infection (Figure 4). Spike areas that later developed FHB symptoms were first detected at 9 dpi, and thus up to four days earlier than by visual rating. A reliable detection of infection was achieved starting at 10 dpi. In addition, LDA was able to successfully differentiate between flowers and infected areas, unlike SVM and the combination of LDA and DC, but provides only a soft classification, which requires manual decision via an expert to set class-specific thresholds to be comparable to the hard classification of SVM and DC. In the analysis of hyperspectral data, LDA is often used for data preprocessing and reduction of dimensionality [35]. However, the algorithm can also be used for classification. Delwiche et al. [36] were able to show that LDA models can distinguish between healthy and *Fusarium* spp.-infected wheat grains. Compared to visual classification, a precision of over 95% was achieved. Huang et al. [37] used a Fisher linear discriminant analysis, a form of LDA, to detect FHB on winter wheat. A precision of 63–86% was achieved, depending on the angle at which the spikes were recorded.

To conclude, the infection with FHB was detected at an earlier stage than by visual rating. This information can be used to initiate countermeasures at an early stage of infection.

### 3.3. Differentiation between FHB Symptoms and Maturity

The spectral signature of senescent plant tissue is very similar to that of FHB-infected spike areas, as the plant tissue there also dies as the infection progresses [5]. Nevertheless, differences were found in the range of 420–600 nm, whereby the reflectance values of senescent tissue up to 540 nm were similar to those of healthy tissue (Figure 2). We tried to improve the precision of classifications by applying a channel selection. For this purpose, the range of 400–800 nm was chosen, as the reflectance values showed discrepancies in this spectrum. However, there were no significant changes resulting from the channel selection. Regarding the differentiation of healthy and FHB-infected wheat grains, it is reported that a focus on the NIR range of 900–1750 nm enables good classification results (summarized in [36]). The spectral signatures in Figure 2 also showed differences for the 940–1000 nm range. However, to study senescent spikes for this wavelength range, a hyperspectral sensor that covers the entire spectrum from 900–1750 nm seems necessary.

### 3.4. Validation of Hyperspectral Imaging by Molecular Analysis

For some sampled spikelets showing or resembling FHB symptoms, molecular detection was carried out by PCR for testing whether they were infected with FHB. Samples were taken at 21 dpi, one day after finishing the hyperspectral measurement series. At this time, advanced maturity was evident on the spikelets, which partially made visual differentiation of senescent spikelets to FHB symptoms difficult. This could explain why FHB was not found in one of the samples from the controls inoculated with FHB. However, since molecular detection was not performed for the entire spikes, it cannot be excluded that they were not infected by FHB at all. However, when entire spikes are tested in PCR, too many inhibitors are present that affect the reaction. Di Pinto et al. [38] report that compounds such as polysaccharides or humic acids often cannot be completely removed by DNA extraction and can interfere or even completely inhibit DNA polymerase activity. In addition, even if a whole spike is positively detected, it is still unclear whether the infected area is on that side of the spike that was also captured by the hyperspectral sensor. A similar problem is reported by Thomas et al. [39], who found a correlation of over 0.72 between the PCA-based disease severity estimation and the qPCR-based measurements of powdery mildew DNA on barley. When infection with powdery mildew was high, the values differed, as powdery mildew was only present and could be detected by hyperspectral imaging in the epidermis layer of the leaf, while qPCR measures the total amount of DNA of the leaf, also including non-infected lower layers.

Except for the spikelet “Control + *Fusarium* 9-4”, a band representing DNA of *Fusarium* spp. was present in all examined spikelets of the treatments inoculated with *Fusarium* (Figure A1). Possibly, the extracted DNA sample of this spikelet contained too many PCR inhibitors, which interfered with the PCR reaction. DNA of *Fusarium* spp. was also detected in the spikelet “Control 1–4”, probably due to natural infection by spore flight. This spikelet was also selected in the training data for the class “Healthy” because the evaluation of the hyperspectral images was performed before molecular pathogen detection due to complications with PCR by non-specific primers. Since PCR is a very sensitive detection method, it is possible that infection was at a very early stage, and thus did not yet cause a measurable change in the image data. This is consistent with the findings of Pane et al. [40], where Tracheofusariosis of wild rocket was detectable by qPCR at 1 dpi, while a detection with HSI was only possible as early as 5 dpi. In addition, it is not known where on the spikelet the infection was located, and thus whether this area is visible in the hyperspectral images. In the training data of the class “Symptom”, the spikelet “Control + *Fusarium* 9-4” was also selected. Thus, gel electrophoresis proved that areas of the spikes were assigned to the wrong classes and provided as training data to the SVM and DC classification algorithms. However, these spikelets represented only a small proportion of the respective training data. The selection of training data by visual assessment, such as visual monitoring, is affected by subjective influence and prone to error [15,41]. This highlights the need for a reliable, automatic pathogen detection.

### 3.5. Evaluation of the Classification Methods

Comparing the visual pixel classification of the reference with the classifications of SVM and DC, the lowest number of pixels was assigned to the class “Maturity” in all variants, as the evaluations were based on the hyperspectral images at 13 dpi. At this time, maturity was not yet predominant and a visual distinction between symptoms and maturity was still possible. On the other hand, more pixels were assigned to the class “Symptom” by SVM and DC than by the reference. While visual classification of pixels for the reference is only based on information of the VIS spectrum, the machine learning algorithms can additionally access the data of the NIR range. Even more importantly, the algorithms are able to detect much finer differences in the highly detailed HSI spectra than humans can by eye in the color shades of an RGB image. Thus, it is possible that the algorithms classified more pixels as symptomatic because they could detect an infection earlier. In this case, additional pixels, located around visually recognizable symptomatic areas, would be labeled as symptomatic by the classification algorithms, too. These pixels were not visually recognizable until subsequent days’ images. To verify this assumption, a confusion matrix, which evaluates the precision of classification algorithms by testing the matches between the classification of the individual pixels and the reference, could be generated. Thomas et al. [20] applied this method to evaluate the precision of their algorithms.

Considering spikes that were classified by the reference as well as the SVM and DC algorithms, it is evident that both supervised methods regularly mislabeled flower areas as symptoms (Figure 6). This could explain the increased assignment of pixels to the class “Symptom” (Table 1). SVM and DC had mainly matched classifications. However, differences in the differentiation between senescent and symptomatic plant tissue were also observed. By adjusting the algorithm parameters, attempts were made to improve the segregation of the classes “Maturity” and “Symptom”, as well as the correct classification of the flowers. Additionally, the use of a channel selection was tested, but no significant optimization was achieved. Bauriegel et al. [23] tested early detection of FHB with HSI combined with principal component analysis (PCA) for different development stages of wheat. They were unable to successfully differentiate healthy and infected spike tissue beginning at the flowering stage and onwards. Tendentially fewer pixels were assigned to the classes “Flower” and “Maturity” by the algorithms than by the reference. However, overall, there was great similarity in pixel assignment to the classes by the reference, SVM and DC. In addition, the validation of the classifications by SVM and DC with the PCR results showed that FHB was reliably detected. Mahlein et al. [42] reported an FHB identification accuracy of 78% with HSI data in combination with SVM classification. The measurements were performed on FHB-inoculated or non-inoculated spikelets of wheat at 3 dpi. At later stages of infection (12, 17 and 21 dpi), the classification accuracy increased to 100%, before it decreased at 30 dpi due to senescence of non-inoculated spikelets. Despite further potential for optimization in terms of differentiation between flowers, symptoms and senescent plant tissue, both SVM and DC enabled earlier pathogen detection compared to visual monitoring and satisfying agreement with the reference.

### 3.6. Visual Monitoring of FHB Symptoms

No significant differences between the treatments were found for FHB incidence, FHB severity and FHB index at both 13 and 17 dpi. These findings are surprising considering the treatments’ values in the corresponding graphs. For example, the average FHB incidence of “Control + *Fusarium*” at 17 dpi was 73.33%, while “Control” at 17 dpi with 0% showed no infection with FHB (Figure 9). This could be due to the small sampling size with three replicates per treatment. Originally, a sampling size with five replicates was intended, but for the HSI it was necessary to place two pots per treatment in a measuring frame, exposing them to different conditions. Therefore, they were not taken into account for the evaluation of the monitoring data. As the values of FHB incidence, FHB severity and FHB index did not follow a Gaussian normal distribution, non-parametric tests had to be chosen for the comparison between the treatments. Parametric tests, on the other hand, have greater statistical power and can thus more easily detect existing significances between groups [43].

Even without apparent significant differences, the treatments show tendencies that can be compared. “T16”, “T23”, “CRP1104” and “HG77” had a low FHB incidence and FHB severity, although they were not inoculated with *Fusarium* spp. (Figure 7, Figure 8, Figure 9 and Figure 10). The infection was likely initiated by natural infection. Between 13 and 17 dpi, FHB incidence, FHB severity and FHB index did not change for these treatments, which indicates a good effect of the three fungal (T16, T23, CRP1104) BCAs and the bacterial one (HG77). All of them were able to prevent the further spreading of the pathogen within the spike and the distribution to further spikes within the respective treatments. A considerable antagonistic potential of T16 and T23 was already found when they were investigated for the interactions with *F. moniliforme* [44] and *F. graminearum* [45] in dual culture and tests on volatile activity. The in vitro studies demonstrated potent inhibitory properties on mycelial growth, sporulation, conidia germination and the length of germ tubes [44,45].

The treatment “Skyway Xpro + *Fusarium*” showed a clearly lower FHB index compared to the other treatments inoculated with FHB at both 13 and 17 dpi. However, considering the FHB incidence at 13 dpi, CRP1104 was as effective (and at 17 dpi almost as effective) as the fungicide in reducing the number of infected spikes. Similar results were achieved by Xue et al. [46], who evaluated the efficacy of *C. rosea* strain ACM941 against FHB in wheat compared to a chemical-synthetic fungicide containing tebuconazole. Under greenhouse conditions, the foliar application of ACM941 two days before pathogen inoculation significantly reduced the number of infected spikelets by 64% compared to the untreated control. At 13 dpi, the bacterial BCA G308 was less effective and showed an FHB incidence almost the same as the BCA-untreated “Control + *Fusarium*”. This trend was also visible for both bacterial antagonists for FHB incidence at 17 dpi, where the values were highest compared with the other variants treated with BCAs and inoculated with FHB. In contrast, at 17 dpi, HG77 and G308 were able to reduce FHB severity better than the other BCAs. Accordingly, Taheri et al. [47] found an endophytic *B. subtilis* strain CB2, which reduced spore germination of the fungus growing on CB2 pre-treated wheat seeds, as well as infection with FHB and the Fusarium crown rot severity of wheat in the greenhouse. Similarly, strains of *P. fluorescens* performed well in the greenhouse and field trials, showing a good biocontrol efficacy against FHB and Fusarium root rot [48,49].

The results suggest that a foliar application of all tested fungal and bacterial BCA strains five and two days before an artificial inoculation of the spikes with *Fusarium* spp. reduces FHB infection, but only to a lesser extent than the chemical fungicide Skyway Xpro. Furthermore, the fungal BCAs seemed to be more effective than the bacterial BCAs, at least in terms of FHB incidence. A combined application of fungal and bacterial BCAs could improve their efficacy, as reported by Karuppiah et al. [50] in a greenhouse trial. Application of both *T. asperellum* GDFS1009 and *B. amyloliquefaciens* 1841 to the potting soil of wheat plants prior to the inoculation with *F. graminearum* resulted in a higher reduction of root rot compared to the control and single treatments. In our study, only Tween 20 and Break-Thru S 301, as wetting and adhesive agents, were added to the applied BCA suspensions. Thus, the spores and bacterial cells were sprayed without any other additives that might shield them from UV radiation or desiccation. Even under such circumstances, the more resistant spores of the fungal BCAs likely had a better chance of establishing themselves on and within the spikes following application, compared to the less robust bacterial cells. This resulted in a lower efficiency of the bacteria regarding the reduction of pathogen spread between different spikes of one treatment (FHB incidence). After successful establishment, however, they were also able to effectively reduce pathogen spread within the spikes (FHB severity).

## 4. Materials and Methods

The trial was conducted in the vegetation hall of the Institute of Phytomedicine at the University of Hohenheim, which consists of a glass-enclosed indoor space with an adjacent outdoor area.

The following variants were investigated, each with and without inoculation of *Fusarium* spp.: Control, fungicide Skyway Xpro (Bayer CropScience Deutschland GmbH, Monheim, Germany), *Trichoderma* sp. T10, *T. harzianum* T16, *T. asperellum* T23, *C. rosea* CRP1104, *B. subtilis* HG77 and *P. fluorescens* G308. The BCAs were obtained from the isolate collection from the Department of Phytomedicine of the University of Hohenheim (Stuttgart, Germany). Each treatment comprised five pots, which also represented the replicates. The treatments that were artificially inoculated with *Fusarium* spp. are labeled with “+ *Fusarium*”.

### 4.1. Plant Material

Spring wheat cv. ‘Servus’ (Hauptsaaten, Köln, Germany) was sown 3–4 cm deep in pots (20 cm height × 15 cm width × 15 cm length) filled with a mixture of equal parts of Classic Profisubstrat CP Topf (Einheitserde Werkverband e.V., Sinntal-Altengronau, Germany) and damped garden compost. For each pot, 20 seeds were used. This wheat cultivar was chosen because of its high susceptibility to FHB, with a score of 6, while being less susceptible to other leaf pathogens according to the official classification of the Federal Plant Variety Office in Germany [51]. The plants were placed on trollies to be moved on rails between the indoor and outdoor areas, and initially cultivated within the indoor area of the vegetation hall until they reached the two-leaf stage (BBCH 12) to protect them from damage by weather and birds. Afterwards, the emerged plants were moved outdoors. After reaching the three-leaf stage (BBCH 13), the number of plants was reduced to six per pot to ensure uniform tillering and stock density. Fertilization was divided into three applications with a total nitrogen quantity of 220 kg N/ha, which converted to the pot area was 0.495 g N/pot. For the first application (28 June 2022), Compo Blaukorn classic (Compo Expert GmbH, Münster, Germany) was chosen to supply the spring wheat not only with nitrogen (12%) but also with phosphorus (8%), potassium (16%), magnesium (3%), sulfur (9%) and various trace elements. Here, 1.375 g/pot was used, equivalent to 0.165 g N/pot, and the fertilizer was spread as granules on the surface of the substrate. The other two fertilizer applications (11 July and 2 August 2022) were performed with ammonium nitrate (NH_4_NO_3_). For each pot, 0.47 g of NH_4_NO_3_ was dissolved in 100 mL of water and evenly distributed on the substrate (0.165 g of nitrogen per pot). In order to protect the plants from infestation of aphids, cereal leaf beetles and other insect pests, they were treated with the insecticide Biscaya (Bayer CropScience Deutschland GmbH, Monheim, Germany) with 1 mL/L of water on 8 July 2022. To prevent bird predation on the ripening spikes, wooden frames were built on the trollies and bird protection nets were mounted on top before the inoculations of the plants started.

### 4.2. BCAs—Cultivation and Inoculation

The fungal BCAs were cultivated on solid glucose medium 7 (GM7), which was prepared according to the recipe of Karlovsky [52], with minor modification. For one liter, 20 g of agar, 1 g of KH_2_PO_4_, 0.5 g of MgSO_4_ × 7 H_2_O, 0.05 g of CaCl_2_ and 0.01 g of FeCl_3_ × 6 H_2_O were dissolved in 900 mL of deionized water, pH was adjusted to 5.5 and then autoclaved at 121 °C and 1 bar for 20 min. Due to heat sensitivity, 3 g of L-asparagine, 0.001 g of thiamine and 20 g of D (+)-glucose monohydrate were dissolved separately in 100 mL of deionized water and sterilized with a vacuum filter before being added to the 900 mL autoclaved medium after cooling to approximately 60 °C. Potato dextrose agar (PDA) plates, well covered with the fungal sporulating BCA cultures, were used to prepare the inoculum for the GM7 medium. Here, 10 mL of 0.01% Tween 20 (MP Biomedicals, Fisher Scientific GmbH, Schwerte, Germany) was pipetted per plate before the biomass was carefully scraped off with a spatula and transferred to a Falcon tube. From this suspension, 0.5 mL was spread on the surface of a GM7 plate and sealed with air-permeable adhesive tape. Incubation of the plates took place at 25 ± 2 °C and in natural light/dark conditions, avoiding direct sunlight for approximately 14 days.

To prepare the BCA inoculum for the wheat plants, 2 to 5 plates from 14-day-old cultures of the respective BCAs were used, depending on the amount of sporulation. Here, 2 × 25 mL of an aqueous solution consisting of 0.01% Tween 20 was added to each plate, mycelium and spores were scraped off and the suspension was filtered through four layers of cotton gauze (Paul Hartmann AG, Heidenheim, Germany). The spore concentration was determined using a Fuchs–Rosenthal counting chamber, adjusted to 1 × 10^7^ spores/mL with 0.01% Tween 20, and then 200 μL/L of Break-Thru S 301 (Alzchem Group AG, Trostberg, Germany) was added. The bacterial BCAs were multiplied and provided by Bio-Protect GmbH (Konstanz, Germany). The culture medium for HG77 contained, per liter, 25 g of sucrose, 2.5 g of Hakaphos green (Compo Expert GmbH, Münster, Germany), 2.7 g of KH_2_PO_4_ and 5.3 g of K_2_HPO_4_. G308 was cultured in Luria-Bertani (LB) medium (per liter, 10 g of tryptone, 5 g of yeast extract, 10 g of NaCl). After propagation, the bacterial liquid cultures were stored at 8 °C in the refrigerator. Before application, they were diluted with the aqueous solution of 0.01% Tween 20 to the final concentration of 1 × 10^8^ cfu/mL, and 200 μL/L of Break-Thru S 301 was added. All BCA suspensions were stored at 4 °C until the evening application.

The Inoculation of the wheat plants with the BCAs was performed 7 and 2 days before pathogen inoculation at a spray rate of 20 mL/pot, using a spray chamber and a chromatography atomizer. Seven days before inoculation with *Fusarium* spp., plants of both fungicide treatments were sprayed with 20 mL/pot of Skyway Xpro (2.5 mL/L). Skyway Xpro (75 g/L of bixafen, 100 g/L of prothioconazole, 100 g/L of tebuconazole) was used according to the instruction manual, with only one application. Control treatments were sprayed with 0.01% Tween 20 + 0.02% Break-Thru S 301 on both application dates, as well as the fungicide treatments 2 days before pathogen inoculation. Subsequently, the spikes were equally moistened with the same volume of water per pot three times on each of the following two days by a hand-held sprayer.

### 4.3. Cultivation of Fusarium spp. and Inoculation

To simulate a natural FHB infection, a mixture of isolates of *F. graminearum* and *F. culmorum* was used in this trial. Spore production of *Fusarium* isolates was performed in carboxymethyl cellulose (CMC) liquid medium, containing, per liter, 15 g of carboxymethylcellulose low viscosity, 1 g of yeast extract, 1 g of NH_4_NO_3_, 1 g of KH_2_PO_4_ and 0.5 g of MgSO_4_ × 7 H_2_O. CMC liquid medium was autoclaved in Erlenmeyer flasks at 121 °C and 1 bar for 20 min.

As an inoculum, carrot agar (CA) medium (20 g of agar, 500 mL of carrot juice and 500 mL of deionized water per liter, pH 4.5) in petri plates (Ø 9 cm) containing the respective *Fusarium* cultures was cut into small plugs, and the plugs from one complete petri plate were added to 1 L of CMC medium in an Erlenmeyer flask. After inoculation, the flasks were sealed with paper plugs and loosely placed aluminum foil to allow air exchange. Liquid cultures were then incubated on a shaker at 150 rpm and 25 ± 2 °C with natural light/darkness conditions for 18 days until sufficient spores were found.

Conidia were harvested by first filtering the liquid cultures through a kitchen sieve to separate agar plugs and mycelium from liquid medium and spores. The solid residues in the kitchen sieve were then washed in deionized water, stirred with a spatula and filtered again through the sieve to transfer the remaining spores into the filtrate as well. Afterwards, the spore suspension was filtered through two layers of cotton gauze to remove smaller mycelium pieces and then centrifuged at 8000 rpm and 25 °C for 20 min. The supernatant was discarded, and the spore pellets were resuspended in the remaining CMC medium and stored at −20 °C until inoculation.

Prior to the inoculation of the wheat with *Fusarium*, 350 mL of spore suspension was thawed from each of the four isolates. After thawing, the suspensions were cloudier than before freezing because solid structures had formed again. Therefore, the spore suspensions were filtered again with two layers of cotton gauze and the filtrate was centrifuged at 8000 rpm and 25 °C for 20 min. The supernatant was discarded, and spore pellets were resuspended in 0.01% Tween 20. Spore concentration was determined for every isolate separately using a Fuchs-Rosenthal counting chamber and brought to the concentration of application of 1 × 10^6^ spores/mL by dilution with 0.01% Tween 20. Before application, a mixture containing equal parts of all four *Fusarium* isolates was prepared and 200 μL/L of Break-Thru S 301 was added. Inoculation with FHB was carried out at full flowering (BBCH 65) in the early evening to ensure successful inoculation. Each pot was sprayed from all sides with 20 mL of spore suspension using a chromatography atomizer. The treatments without *Fusarium* were sprayed only with 0.01% Tween 20 + 0.02% Break-Thru S 301. For four days after inoculation, wheat spikes were sprayed equally with the same volume of water per pot three times a day to increase the chance of successful infection using a hand-held sprayer.

### 4.4. Monitoring of FHB Symptoms

After the first visible symptoms appeared, visual ratings were carried out to assess the FHB infection. Both FHB incidence and severity were evaluated, and the FHB index was determined. While FHB incidence describes the proportion of spikes examined that showed symptoms of FHB infection, FHB severity represents the proportion of spikelets of a spike that were infected by the pathogen. The FHB index is based on both values and was calculated with the formula of Góral et al. [53]:

$$FHB\;index(\%)=\frac{FHB\;incidence\left(\%\right)\times FHB\;severity(\%)}{100}$$

Monitoring was carried out for all pots and treatments at 13 and 17 dpi with FHB. FHB incidence was determined on five spikes per pot and FHB severity on three spikes per pot. In both cases, spikes were randomly chosen. For the HSI, before performing the monitoring, two pots of each treatment (pots 4 and 5) were randomly selected and fixed in a measuring frame, where they were exposed to different conditions than pots 1–3. Therefore, the spikes of pots 4 and 5 were also assessed in the evaluation of FHB infection but were not considered and not equated with the other three replicates (pots 1–3).

### 4.5. Hyperspectral Measurement Series

**Figure 11:**
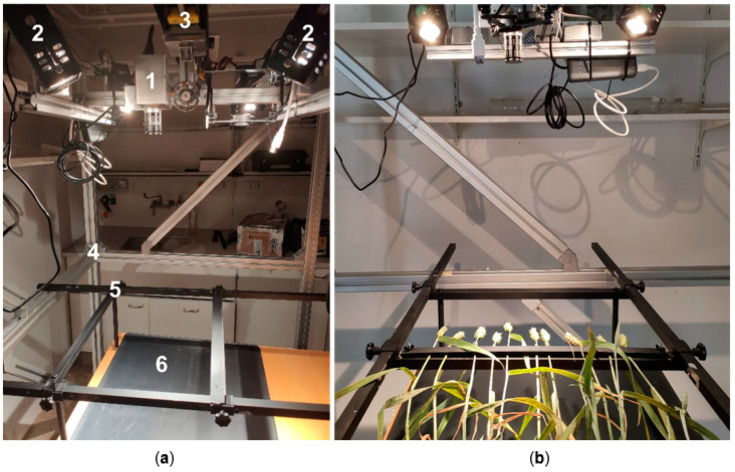
Measuring stand for hyperspectral recordings in the darkened laboratory: (**a**) Components of the measuring system with (1) hyperspectral sensor, (2) halogen lamps, (3) guide rail of the linear motor, (4) metal frame, (5) measuring frame and (6) table. (**b**) The frames with the fixed spikes were placed horizontally on the table for measurements.

For the pots 4 and 5 of all treatments, the number of spikes was reduced to five spikes per pot by cutting off the surplus stalks close to the ground, and the remaining spikes were each fixed in a measuring frame by a metal bar padded with foam. The laboratory measurements were carried out on a measuring stand with the Corning microHSI 410 hyperspectral sensor (Corning Inc., New York, NY, USA), which measures the reflection in a spectrum of 402.76–994.54 nm at a distance of 2 nm and calculates the reflectance values. The sensor and four halogen lamps (Malvern Panalytical Ltd., Malvern, UK) were attached to a linear motor and guide rail, which moved them horizontally (Figure 11).

The halogen lamps, which were switched on 30 min before starting the measurement to avoid fluctuations in the spectrum of their emitted light, were the only light source in the measurement laboratory during the recordings. The recordings were produced by use of the fluxTrainer Pro 4.6 software (LuxFlux GmbH, Reutlingen, Germany) as control and recording software for the affiliated hardware. To ensure comparability of the measurements, calibration was performed with the dark and white references (SG 3151, SphereOptics GmbH, Herrsching am Ammersee, Germany) and the mean value from 100 frames was used in each case.

The measurements of the spikes were then carried out. For the measurements, the frame was rotated by 90° and the spikes were placed horizontally under the hyperspectral sensor with the same distance from the sensor. Due to the horizontal position of the spikes, a larger spike area could be observed. In order to avoid differences by the time of day between the recording days, the different treatments were always measured at the same time. Starting at 6 dpi, hyperspectral images of the treatments were taken daily. The measurement series was finished at 20 dpi, as the spikes showed progressive maturity.

### 4.6. Evaluation of the Hyperspectral Images

The software fluxTrainer Pro 4.14.1.5 (LuxFlux GmbH, Reutlingen, Germany) was used to evaluate the hyperspectral images. It acts as a basic tool to apply various data analysis methods, such as LDA, SVM and DC, for the analysis of the pre-processed masked hyperspectral images without background. For all evaluated hyperspectral images, stalks and leaves were manually marked and cut out, as otherwise these would interfere with the classification of the spikes. Spike areas were manually assigned to the created classes “Healthy”, “Flower”, “Symptom” and “Maturity” as training data for pathogen detection with the supervised algorithms. To ensure comparability, the training data were identical in all projects. Non-symptomatic spike areas of the hypercube “Control” at 9 dpi were assigned to the class “Healthy”. The flowers were excluded and assigned to the class “Flower” instead. Only spike areas from “Control + *Fusarium*” at 20 dpi were added to the class “Symptom” if they were visually infected by FHB. The training data of the class “Maturity” originated from the hyperspectral image “Control” at 20 dpi. During classification, the pixels were converted by the machine learning algorithms into the color of the assigned classes: “Symptom” red, “Healthy” green, “Flower” yellow and “Maturity” blue.

For the treatments “Control + *Fusarium*”, “T10 + *Fusarium*”, “T16 + *Fusarium*” and “T23 + *Fusarium*”, the hyperspectral images of all measurement days were evaluated with the fluxTrainer. For each treatment, three representative and adjacent spikes, which showed clear FHB symptoms at the end of the measurement series, were selected for evaluation.

### 4.7. Quantitative Evaluation of the Classification Methods

For objective evaluation of the classification methods SVM and DC, their classification of pixels was compared with the manual classification of the pixels, the reference. The evaluation was carried out with the image processing software GIMP Version 2.10.12 (The GIMP Development Team) by loading the hyperspectral images of the respective treatments recorded at 13 dpi as pseudo-RGB images and classifying each pixel into one of the four classes, “Healthy”, “Flower”, “Maturity” and “Symptom”, by means of manual coloring by the experts M.L. and M.R.. The pseudo-RGB images were generated from the respective hyperspectral images. To ensure comparability, the same processing chain was used as for the classification methods and the same spikes were evaluated. The total pixels assigned to the respective classes and the pixel proportions by the different classification methods were compared with each other.

In addition to the comparison of the pixel proportions assigned to the four classes, the images of the reference and those of the classifications by SVM and DC were also compared. This was performed to check not only whether the pixel proportions were similar, but also whether the spike areas were assigned to the same classes for all the classification methods.

### 4.8. Molecular Analysis with PCR

Several spikelets per treatment were taken as samples and stored at −20 °C until molecular analysis. While symptomatic spikelets were selected for the treatments inoculated with FHB, this was not possible for most of the treatments without *Fusarium* inoculation. In this case, necrotic or conspicuous spikelets were sampled. The samples were ground using liquid nitrogen. DNA extraction was performed according to the protocol of Liu et al. [54], whereby in the last step, the isolated DNA was resuspended with H_2_O_pure_ instead of TRIS-EDTA. The concentration and quality of the isolated DNA were checked, and PCR was performed with the C1000 Touch Thermal Cycler (Bio-Rad Laboratories Inc., Hercules, CA, USA) using Taq polymerase. The reaction mix is shown in Table 2.

PCR was performed using the primer pair “TEF-Fu3 f” and “TEF-Fu3 r” [55] with the parameters shown in Table 3. The primers are genus-specific for *Fusarium* spp. binding to the gene TEF-1α. Furthermore, it was proven that DNA of the BCAs was not amplified by the primers. In addition to the DNA of the spikelet samples, a no-template (NT) control and a positive control were included. For the positive control, DNA was extracted from mycelium of the four *Fusarium* isolates used, mixed and added to the PCR reaction.

After completion of 40 cycles, the PCR products were determined by gel electrophoresis. Therefore, 6 μL of PCR product was mixed with 1 μL of 6x Loading Dye and pipetted into the pockets of a 2% agarose gel. Then, 6 μL of GeneRuler 100 bp (Thermo Fisher Scientific Inc., Waltham, MA, USA) was used as a DNA ladder. The gel was then run at 100 volts for 45 min, followed by staining in an ethidium bromide bath for 10 min and then, after 20 min in a water bath, analyzed.

### 4.9. Data Analysis

Evaluation of the monitoring data for FHB incidence and severity of replicates 1–3 (pots 1–3) was planned to be performed by analysis of variance for the data of the two monitoring dates, separately. R Version 4.2.2 (Posit Software, Boston, MA, USA) was used as statistical software. The prerequisites of the analysis of variance are the homogeneity of the variances and the normal distribution of the data. The homoscedasticity could be proven with the help of the Levene test from the “car” package. It was also confirmed visually with the help of residual plots. However, the Shapiro–Wilk test and a QQ plot proved that there was no normal distribution. Therefore, non-parametric tests had to be used. Various non-parametric tests for independent samples, for example the Kruskal–Wallis test or the Wilcoxon test, could not be applied due to ties within the data. Instead, a one-factorial general linear regression model was generated with the “Logistic Model” based on the existing data. This was carried out with the function “glm”. “Least Square Means” was then used to test for significant differences between the treatments by the function “lsmeans” from the package “emmeans”. The alpha error accumulation was adjusted according to Bonferroni. Graphs were created in Excel (Microsoft Corporation, Redmond, WA, USA).

## 5. Conclusions

Healthy spike tissue was characterized by very low reflectance values in the range of 500–680 nm, as these wavelengths are used for photosynthesis. Flowers, mature and FHB-infected spike areas were limited in their photosynthetic activity and showed higher reflectance for 500–680 nm than healthy spike tissue. The differences in the spectral signatures for differentiating between flowers, senescent tissue and FHB infection were small. While flowers consistently had the highest reflectance values in the range of 450–740 nm, senescence and FHB symptoms could be distinguished primarily on the basis of the 420–600 nm spectra, as well as 900 nm and above.

FHB infection was first detected by the LDA at 9 dpi, or reliably at 10 dpi, which means four days before visual identification (13 dpi). With SVM, the first detection was at 11 dpi, while DC with preceding LDA showed the later symptomatic areas at 10 dpi. Pathogen detection by K-Means clustering did not occur earlier than visual detection with the pseudo-RGB images. Based on these results, a monitoring system enabling a rapid and precise identification and quantification of FHB infections at the field level can be developed and used in practical agriculture. This allows conducting appropriate countermeasures quickly and in a targeted manner.

Molecular PCR analysis was used to verify the selection of training data and the classifications of the machine learning algorithms. The results showed that a small proportion of the manually selected training data was misclassified. Although this probably did not have a significant impact on the spectral signatures or the classification algorithms, it highlights the high relevance of automatic FHB detection to avoid subjectivity and errors in visual rating.

Concerning accuracy, both supervised methods, SVM and DC, performed better than the unsupervised K-Means clustering, as K-means clustering was not able to distinguish between flowers, maturity and symptomatic areas. SVM and DC showed high agreement compared to the manually evaluated reference as well as to the results of PCR analysis. However, differentiation between FHB-infected areas and flowers, as well as between FHB-infected areas and senescent plant tissue, still needs further optimization.

The tested BCAs had no significant differences between the treatments concerning their efficiency in reducing FHB infection, but all of them tendentially seemed to have a reducing effect, with a greater effect of the fungal than the bacterial BCAs. However, without a supporting formulation of the fungal spores and bacterial cells, their efficiency is still lower than that of a chemical-synthetic fungicide.

## Figures and Tables

**Table 1:** Quantitative comparison of pixel allocation by reference, SVM and DC for the variant “Control + *Fusarium*”. The classification is based on three spikes at 13 dpi.

	Reference		SVM		DC	
Class	Count	Percentage	Count	Percentage	Count	Percentage
Healthy	50,438	91.52	49,584	89.97	47,921	86.96
Flower	1007	1.83	196	0.36	747	1.36
Maturity	181	0.33	3	0.01	12	0.02
Symptom	3484	6.32	5327	9.67	6430	11.67
Total	55,110	100.00	55,110	100.00	55,110	100.00

**Table 2:** PCR mix with Taq DNA polymerase for one reaction.

Reagents	Volume per Reaction (µL)
H_2_O_pure_	13.00
10× Taq Buffer	2.50
MgCl_2_	2.50
dNTPs	2.50
Primer forward	1.25
Primer reverse	1.25
Taq DNA Polymerase	1.00
DNA	1.00
Total	25.00

**Table 3:** Parameters for PCR reaction with the primer pair “TEF-Fu3”. Steps 2 to 5 are cycled 40×.

Step	Temperature (°C)	Duration (min:s)
Denaturation	94	5:00
Denaturation	94	1:00
Annealing	58	1:00
Elongation	72	2:00
Hybridization	72	5:00
Storage	4	∞

## Data Availability

Due to the large size of the hyperspectral datafiles, the datasets of the study are available upon request to the corresponding author.
